# Neuropsychological exponents for the driving ability in remitted bipolar patients

**DOI:** 10.1186/s40345-021-00247-z

**Published:** 2022-01-23

**Authors:** Piotr Joachimiak, Krystyna Jaracz, Jan Jaracz

**Affiliations:** 1 Department of Adult Psychiatry, University of Medical Sciences, Poznan, Poland; 2 Department of Neurological Nursing, University of Medical Sciences, Poznan, Poland; 3 Klinika Psychiatrii UM w Poznaniu, ul. Szpitalna 27/33, 60-572 Poznan, Poland

**Keywords:** Bipolar disorder, Cognitive impairment, Driving abilities, Mood-stabilizing drugs

## Abstract

**Background:**

Bipolar disorder (BD) is associated with cognitive deficits regardless of the phase of the disease. Medications used in treatment are an additional factor that may affect cognitive performance. Poor cognitive performance can significantly affect a patient's ability to drive.

**Aim of the study:**

This study aims to explore cognitive functions relevant for safe driving in the group of remitted bipolar patients.

**Method:**

Patients with BD in remission (n = 33) and healthy volunteers (n = 32) were included. Selected psychometric tests for drivers were carried out using computer software: called Specialistic Diagnostic Platform (SPD): The Cross-over Test (COT) version with free tempo (COT-F) and tempo of 50 tasks per minute (COT-50) and the Signal Test (ST). Moreover, the following neuropsychological tests were used: Rey Auditory Verbal Learning Test (RAVLT), Stroop Color-Word Test (SCWT) part A and B, and Trail Making Test (TMT) version A and B.

**Results:**

In comparison with healthy controls bipolar patients in remission had poorer outcomes for some cognitive parameters and longer reaction times in both tests for drivers and neuropsychological tests. Additionally, we found a significant correlation between the time of performance of neuropsychological tests and the time of psychometric tests for drivers.

**Conclusion:**

Patients with BD performed worse in several cognitive domains assessed by tests for drivers and neuropsychological tasks. These deficits can affect the speed of the patient's motor reactions while driving.

## Introduction

Bipolar disorder (BD) is a recurrent, usually lifelong, disease that has a negative impact on social and professional functioning, commonly resulting in disability. One of the factors contributing to these negative consequences is cognitive dysfunctions including impairment of verbal memory, attention, executive functions in bipolar patients, which have been found during mania/hypomania and depression, all of which are also seen in periods of remission (Martinez-Aran et al. 2004; Cavanagh et al. 2002; Robinson et al. 2006; Mann-Wrobel et al. 2011; Arts et al. 2007; Bourne et al. 2013). A recent systematic review and meta-analysis of thirty-six studies identified deficits in several domains of executive function deficits including set-shifting, inhibition, planning, verbal fluency, working memory, and attention in BD patients (Dickinson et al. 2017). The results of this study indicate that BD I subjects performed worse than healthy controls in all domains. Moreover, BD II subjects demonstrated similar or even greater impairment of selected executive functions.

In everyday life, the correct state of cognitive functions is essential for safe driving. This activity requires, among other things, efficient attention, working memory and reaction speed. In Germany, in a group of 1497 psychiatric patients, 67% reported having a valid driver's licence. Seventy-seven percent of them (versus 92% of a control group) reported regularly using their cars (Brunnauer et al. 2016). A significant proportion of people affected by BD have a driving licence and use a car daily. Regardless of the impact of the disease itself, psychotropic drugs, used in acute states and prophylactic treatment, may affect cognitive functioning. The question of whether a remitted bipolar patient who is on mood-stabilizing medications can drive a vehicle is often raised in everyday clinical practice. However, the answer is difficult due to the small amount of research devoted to these issues. In the drug prescribing information, one can find information suggesting ‘caution’ or the prohibition of driving during pharmacotherapy.

A case–control study performed in the Netherlands found an association between the risk of having a motor vehicle accident and the exposure to anxiolytics and selective serotonin reuptake inhibitors (SSRIs) but not with the use of antipsychotics (Ravera et al. 2011). On the other hand, Miyata et al. (2018) found that partly remitted depressive patients treated with antidepressants (monotherapy and combination) do not differ from healthy controls concerning the performance of Wisconsin Card sorting test, TMT, and Continuous Performance Test. Brunnauer and Laux (2017) found, based on a systematic literature review, that SSRIs and serotonin-norepinephrine reuptake inhibitors (SNRIs) venlafaxine and milnacipran did not affect driving ability.

Results of De Las Cueva's study (2010) indicated that 90% of patients with mental disorders in the acute state do not meet the psychometric conditions for obtaining a driving licence. However, rational pharmacotherapy and a state of remission can improve that situation. The presence of depression is a major cause of driving impairment, while reducing the severity of depression in the course of treatment with antidepressants usually reduces the severity of this problem.

Therefore, it would be interesting to assess the driving ability of bipolar patients in remission and taking mood stabilizers (MS).

In the treatment of BD, the following drugs are recommended: lithium, valproic acid, carbamazepine so-called ‘classic’ or first-generation mood-stabilizing drugs (MSDs) lamotrigine, atypical antipsychotics: olanzapine, quetiapine, clozapine, aripiprazole, asenapine, paliperidone referred to as second-generation MSDs (Grunze et al., 2013). These medications are used in monotherapy and commonly in polypharmacy.

The number of studies devoted to the influence of MSD on the ability to drive vehicles is scarce so far. Laux and Brunnauer (2014) suggest that only 17% of remitted BD patients can be considered unfit to drive. Hatcher et al. (1990) found that patients taking lithium had slower responses in the driving simulator compared to healthy volunteers. A comparison of carbamazepine and oxcarbazepine showed oxcarbazepine was better tolerated and had a lower impact on cognitive function. However, the effectiveness of oxcarbazepine in stabilizing mood is not as well proven as in the case of carbamazepine (Kaussner et al. 2010; Mecarelli et al. 2004). Segmiller et al. (2013) found that 45% of patients with BD treated with lithium or lamotrigine correctly performed psychometric tests for drivers. Lamotrigine was better tolerated than lithium. For this reason, we undertook research aimed at evaluating cognitive function ability to drive of remitted bipolar patients receiving prophylactic treatment.

## Material and method

Thirty-three remitted bipolar patients (twenty two 22 females and eleven 11 males) from the outpatient clinic of the Department of Adult Psychiatry of the Medical University of Poznań and 32 healthy volunteers were included in the study.

The diagnosis of BD was made based on DSM-5 criteria. Clinical data were obtained from medical records and based on a patient’s interview performed by PJ. The mental status examination was assessed before inclusion in the study. We applied generally accepted psychometric criteria of remission in BD that is Hamilton Depression Rating Scale—HAM-D score < 8 and Young Mania Rating Scale YMRS score < 12. The inclusion and exclusion criteria are presented in Table 1.

**Table 1:** Inclusion and exclusion criteria for patients

Inclusion criteria
Patients of both sexes aged 18–65
BD diagnosis in remission (HDRS < 8p, YMRS < 12)
Regular use of recommended mood stabilizers
Patient's consent
Exclusion criteria
Clinically unstable medical conditions
Mental retardation or symptoms of dementia
Electroconvulsive therapy in the last year
Addiction to any psychoactive substances
Current use of sedatives (BDA and derivatives, hydroxyzine, hypnotics) or other sedative (e.g. some allergy, drugs)
Significant vision deficits

*HDRS* Hamilton Rating Scale for Depression, *YMRS* Young Mania Rating Scale

The demographic and clinical characteristics of both groups are shown in Table 2. There were no statistically significant differences in the sex ratio and mean age between study groups.

**Table 2:** Demographic and clinical characteristics of patients and the control group

	Patients n = 33	Control group n = 32	Statistics
Age: mean (SD)	39.5 (11.11)	41.63 (9.63)	t = 0.81, p = 0.424 NS*
Male/female	11 (33%)/ 22 (67%)	17 (53%)/15 (47%)	Chi^2^ = 0.15, p = 0.698 NS**
Education: university/secondary or primary school	18 (55%)/10 (30%)	23 (71%)/9 (18%)	Chi^2^ = 2.09 p = 0.147 NS**
Work activity	18 (55%)	32 (100%)	p < 0.001***
Driving licence (yes)	22 (66%)	30 (93%)	p = 0.011***
Time from obtaining the driving licence (years, mean (SD))	26.5 (8.6)	20.4 (10.2)	t = 2.61 p = 0.011*
Duration of illness years: mean (range)	6.2 (0–23)	–	NA

^*^The t-Student test, **Chi^2^ test, *** Fisher exact test

The control group consisted of healthy volunteers aged 18 to 65, without mental, general medical, neurological and addiction disorders, without severe vision deficits, and also not using hypnotics or sedatives (benzodiazepines, hydroxyzine). Clinical data from control participants was obtained based on a structured interview carried out by PJ.

All subjects from the control group and slightly more than half of the patients group were employed.

Most of the participants had an provisional driving licence. The average time of having held a driving licence was numerically longer in the group of patients (Table 2).

All patients used psychotropic medications because of BD. Most of them were using two 2 or more mood-stabilizing drugs. Only four patients were on monotherapy (quetiapine n = 2, carbamazepine n = 1, lamotrigine n = 1). Details of pharmacological treatment and mean doses of psychotropic drugs are given in Table 3.

**Table 3:** Psychotropic drugs (mean doses) used in the studied patients

	Lithium (908 mg) n = 19	Valproates (1044 mg) n = 8	Carbamazepine (800 mg) n = 2	Quetiapine (265 mg) n = 17	Olanzapine (12.5 mg) n = 7	Clozapine (200 mg) n = 2	Risperidone (1,5 mg) n = 2	Lamotrigine (147 mg) n = 8	Sertraline (67 mg) n = 3	Fluoxetine (20 mg) n = 2	Paroxetine (20 mg) n = 1	Escitalopram (17.5 mg) n = 2	Venlafaxine (196 mg) n = 10	Mirtazapine (27 mg) n = 5)	Bupropion (150 mg) n = 1	Trazodone (75 mg) n = 1
1	✓		✓				✓									
2	✓			✓												
3	✓				✓											
4	✓			✓									✓			
5		✓		✓												
6				✓	✓											
7		✓		✓			✓									
8	✓					✓		✓			✓		✓			
9	✓			✓									✓			
10				✓					✓						✓	
11				✓						✓						
12				✓												
13	✓				✓											
14				✓												
15	✓					✓							✓			
16	✓	✓		✓												
17			✓													
18	✓	✓											✓	✓		
19	✓			✓				✓								
20	✓				✓								✓	✓		
21		✓											✓	✓		
22	✓			✓												
23	✓			✓		✓										
24								✓								
25	✓	✓			✓				✓							
26	✓	✓		✓				✓								
27		✓							✓							✓
28				✓	✓			✓		✓						
29	✓				✓								✓			
30								✓					✓			
31	✓											✓		✓		
32				✓				✓				✓		✓		
33	✓							✓					✓			
N	19	8	2	17	7	3	2	8	3	2	1	2	10	5	1	1

Selected psychometric tests for drivers were carried out by computer software: Specialistic Diagnostic Platform (SPD): The Cross-over Test (COT)—version with free tempo (COT-F) and tempo of 50 tasks per minute (COT-50) and The Signal Test (ST). Additionally, cognitive functions were assessed using Rey Auditory Verbal Learning Test (RAVLT), Stroop Color-Word Test (SCWT) part A and B, and Trail Making Test (TMT) part A and B.

The Crossover Test is a classic method for assessing several psychometric parameters. This test is useful for the assessment of reaction speed, eye-hand coordination, ability to concentrate attention, and speed with perception accuracy. In the version of the test with the imposed pace, we can additionally check the patient's speed of decision-making in situations performed under time pressure and fatigue resistance. The device for carrying out the test consists of a button board with a row of LEDs at the top and a column of LEDs on one side, depending on the patient's right or left-handedness. During the test, two LEDs light up simultaneously—one in a row, the other in a column. The patient's task is to find a button on the board that lies at the intersection of imaginary semi-straight lines running from the lighted diodes. The test consists of two parts: one with a free pace and one with an imposed pace. In the first part, the patient performs a certain number of tasks without time pressure, but with the aim of performing the test as soon as possible. In the second part, the participant has the same number of tasks to perform, but with a response time imposed (e.g. 1.2 s per response). The test calculates the number of correct and delayed responses, as well as the number of incorrect reactions or no response. Empirical studies have shown the accuracy of eye-hand coordination tests using the COT (Horowski 2012).

The Signal Test measures the response time. It consists of two phases: simple response time and choice response time. The reaction time is the time required for a response to the external trigger that occurred (critical). In a simple reaction, one stimulus determines a specific response. The time of this reaction is shorter than the time to react with the choice because it also includes the moment of deciding on a specific response to a specific factor when various triggering factors are presented, for example, different reactions to colours such as red, yellow and so on (Horowski 2012). In the first part of the test, the stimulus does not change and always forces the same reaction. In this way, a simple reaction is measured. In the second part, stimuli are randomly changed and force a subject to think about a specific reaction to a specific stimulus. In this way, the reaction time with choice is measured. In addition to the basic indicators for both parts, such as the average response time, minimum, maximum, and deviation from the average, as well as the number of erroneous reactions, it is possible to estimate the difference indicator (response time with choice minus the simple response time), which, according to Sternberg's theory, reflects the time involved in the decision-making part (stimulus assessment and response control).

The Rey Auditory Verbal Learning Test is used to assess short-term and delayed memory, auditory learning, and distractibility. The Stroop Color-Word Test (part A and B) has been designed to measure the ability to inhibit cognitive interference that occurs when the processing of a specific stimulus feature impedes the simultaneous processing of a second stimulus attribute. This test also measures verbal abilities and attention (Part A), working memory, and executive functions (part B) (Scarpina and Tagini 2017). The results of TMT provide information on visual search, scanning, speed of processing, mental flexibility (shifting strategy), and executive functions (Tombaugh 2004).

### Statistical analysis

Statistical analysis was performed using the software Statistica 12, Statsoft company.

## Results

In the COT at a free rate, the group of patients performed more slowly than the control group. The analysis showed statistically significantly slower simple reactions in patients compared to healthy volunteers. In the COT-50 test at the rate of 50 stimuli/minute, patients were less able to react to the stimulus properly compared to the control group (Table 4). Results of the patients in comparison to the control subjects were worse by ≥ SD in 12.1% (COT-F) and 53.1% (COT-50).

**Table 4:** Results of the psychometric tests—statistically significant differences (*SD* standard deviation; *Mann–Whitney U test)

	Patient group (n = 33)			Control group n = 32			
	Mean	Median	SD	Mean	Median	SD	p
The Cross-Over Test—free rate (COT-F)							
Test time (s)	115.8	104.2	40.4	101.9	98.6	22.3	Z = 2,13 p = 0.03*
Median response time (ms)	1471.5	1340	468.16	1268.6	1261.5	154.39	Z = 2.30 p = 0.02*
The Cross-Over Test—rate of 50 stimuli/minute (COT-50)							
Number of stimuli received	14.6	10	16.7	27.3	25.5	193	Z = 2.97 p = 0.002*

	Patient group			Control group			p
	Mean	Median	SD	Mean	Median	SD	
Rey's Auditory Verbal Learning Test (RAVLT)							
The number of perseverations	4.4	3	3.78	2.3	2	3.01	Z = 2.93 p = 0.003
The number of words in the deferred sample	2.2	2	1.72	1.3	1	1.95	Z = 2.44 p = 0.01

	Patients (n = 21)			Controls (n = 26)			p
Stroop Color-Word Test (SCWT)							
Part A (s)	25.19	25.0	4.6	22.46	22.0	3.84	Z = 2.00 p = 0.045 T = 0.031*
Part B (s)	57.14	14.61	14.61	51.42	9.91	9.92	Z = 0.95 p = 0.341
Trail Making Test (TMT)							
Part A (s)	30.94	28	12.7	24.22	23	8.34	Z = 2.08 p = 0.036
Part B (s)	78.76	65	43.4	58.81	55	22.68	Z = 2.41 p = 0.016

In the Signal Test, there were no significant differences between patients and healthy volunteers.

The patient group performed significantly worse in two parameters of Rey's Auditory Verbal Learning Test: the sum of words perseveration and the number of words in the deferred sample. The patients repeated the same words more often in an attempt to reproduce 15 words. Furthermore, in the last attempt, postponed by 20 min, significantly more words were forgotten by patients in comparison to the control group (Table 4). The proportions of patients for whom these results were worse by ≥ SD than in the controls were 27.3% and 33.3%, respectively.

In the SCWT, the group of patients performed for longer in parts A (2.63 s.) and B (5.72 s.) of the test. However, the difference was only statistically significant in part A (Table 4). In both parts of this test, 28% of the patients' results were worse by ≥ SD compared to the controls’ results.

In the TMT test, the patient's group performed the whole test (both parts A and B) slower than the control group (Table 4). In terms of ≥ SDs, the results were worse for 30.3% and 18.2% of the patients, respectively.

Spearman's rank correlation analyses did not show significant relationships between the patients' ages, duration of the disease, and the performance of TMT-A, and- B and SCWT-A and B. The same was observed in the control group, excluding the disease duration.

However, we found several significant positive correlations between the patients age and performance of COT-F in particular: the median time to complete the test (r = 0.395), the count of mistakes made (r = 0.404), the median response time (r = 0.390), the count of false reaction in COT-50 (r = 0.410) and the time to complete the ST time (5 out of 6 parameters). The count of false reaction in COT-50 also correlated with the duration of the disease (r = 0.355). In the control group, there were no significant correlations between the participants age and performance of COF-F and COT-50 but, positive correlations were found in the ST time (3 parameters).

Time of performance of neuropsychological tests correlated significantly with the time of psychometric tests for drivers (p < 0.05). The time to complete the COT-F with the time of TMT-A (0.546) and TMT-B (0.632) as well as with SCWT- time (0.476). Results of ST also correlated with time of performance of the psychometric test (TMT A x ST median time of simple reaction (0.356), TMT A x ST median time of reaction with choice (0.363).

In addition, male–female differences both within and between groups the differences were also examined. The only within-group differences were found in Rey's Auditory Verbal Learning Test: the number of perseverations (M = 2.39, SD 2.96 vs F = 4.08, SD 3.84, p = 0.046) and the COT-F: the median response time (M = 104.2, SD 27.8 vs F = 112.6, SD 36.9, p = 0.026). The between-groups differences appeared only in the control group. Men performed better in COT-F: the test time (M = 100.2, SD 30.0 vs F = 103.4, SD 8.1, p = 0.049) and the median response time (M = 1206.1 SD 163.9 vs F = 1339.5, SD 109.2, p = 0.014).

In the group of patients treated with lithium, the response time in the COT-F test was longer, on average, 2 s than in the group not receiving lithium. Likewise, in the ST test, the response time in this group was longer by 0.067 s and the total response time by 0.12 s. We found no differences in the results of neuropsychological tests in patients who received antidepressants and those who did not receive such drugs.

## Discussion

The main finding of this study is that remitted bipolar patients performed worse in the COT-F. The response time was significantly longer than in the control group. The COT examines the attention in terms of vigilance and durability, as well as visual-motor coordination, which is responsible for the speed of reaction. In the free tempo version, the group of patients worked slower. The median response time to the stimulus in the group of patients was 1.47 s (median 1.34 s), while in the control group it was 1.27 s (median 1.26 s). The difference is therefore 0.2 s (median 0.08 s).

To determine whether these differences are relevant to driving safely, it is possible to apply simple physics formula presented in Table 5.

**Table 5:** Formula for calculating distance travelled at constant speed per unit of time

$\Delta s=v*\Delta t$	*Δs—distance*
	*v—speed*
	*Δt—time*

According to the data contained in the table, the unit of time 0.08 s and 0.2 s is not negligible when driving a vehicle. A delay in the driver's reaction by just 0.08 s results in travelling another 1 m of the road at the typical speed in the city of 50 km/h (according to road regulations in Poland). With a delay of 0.2 s, there is an even longer distance of almost 3 m. In city traffic, these can be significant distances and determine the scale of a potential accident (Table 6).

**Table 6:** Distance travelled by an object per unit of time at a constant speed

	Speed (km/h)									
	10	20	30	40	50	60	70	80	90	100
Time	Distance									
1 s	2.78 m	5.56 m	8.34 m	11.1 m	13.89 m	16.67 m	19.44 m	22.22 m	25 m	27.78 m
0.2 s	0.56 m	1.11 m	1.67 m	2.2 m	2.78 m	3.3 m	3.89 m	4.44 m	5 m	5.56 m
0.08 s	0.22 m	0.44 m	0.67 m	0.89 m	1.1 m	1.33 m	1.56 m	1.78 m	2 m	2.22 m

The next statistically significantly worse result in the group of patients is the overall time of performing the test with the COT-F. The patient group took 14 s more to perform the whole test (median 5 s) than the control group. This gives an average of 1.6 s (median 1.4 s) on the response in the patient group and 1.4 s (median 1.3 s) in the control group. Differences are also in the order of tenths of seconds, but as it has been shown earlier, it can be important for road safety.

The COT-50 required a response to the task within 1.2 s for each of the 75 tasks. The patient group responded correctly to 19.5% of tasks (median 13.3%) and the control group to 36.4% (median 34.0%). The preliminary analysis shows that the test was difficult even for healthy people. There is a clear disproportion in the count of correct responses between the studied groups. This confirms previous observations of a slower response time in patients suffering from bipolar disorder during remission.

Results of the neuropsychological tests have also shown a decline of cognitive functions in the patients’ group in comparison to healthy people. Statistically significantly worse results in RAVLT point to deficits in verbal memory. While driving, verbal memory seems to be important when reading road signs and signposts and other traffic information. The slower tempo of performance of the SCWT suggests disturbances in the attention function and problems in inhibition of cognitive inference which is a common situation while driving. The slower rate of performance of the TMT test suggests that bipolar patients have problems with visual search, scanning of the external situation, speed of processing of external information, and mental flexibility. These abilities have a significant impact on safe driving Our findings are consistent with the results of numerous previously published studies (Martinez-Aran et al. 2004; Cavanagh et al. 2002; Robinson et al. 2006; Mann-Wrobel et al. 2011; Hsiao et al 2009; Torrent et al. 2006; Xu et al 2012; Ha et al. 2014; Quraishi and Frangou 2002) and meta-analysis (Arts et al. 2007; Bourne et al. 2013).

We did not find too many studies devoted to this issue in the PubMed database. Hetcher et al. (1990) compared the group of patients diagnosed with bipolar disorder in the remission period and used lithium with a group of healthy volunteers using a driving simulator. The group of patients presented a slower reaction to traffic situations.

De Las Cuevas et al. (2010) showed that 83% of patients who were unable to drive a car at the time of the psychiatric diagnosis after 6 weeks of treatment improved their cognitive state, which significantly reduced the risk of a road collision. Among them, 25% could drive vehicles without any obstacles. According to the Laux and Brunnauer study (2014), only 17% of patients with bipolar disorder in remission should be considered unfit to drive.

Our findings also indicate a correlation of neuropsychological and psychometric tests for drivers’ results. This confirms the usefulness of both types of tests in the assessment of cognitive functions relevant to safe driving. Performing these paper-and-pencil neuropsychological tests is often possible in psychologists’ offices.

In the group of remitted BD patients, deficits in cognitive and executive functions which can affect safe driving were found. Previous research indicates that cognitive deficits are independent of the drugs used and are deeper in untreated patients and acute bipolar states (De Las Cuevas 2010; Xu et al. 2012; Ha et al. 2014; Quraishi and Frangou 2002; Dittmann et al. 2008; Mahli et al. 2007; Volkert et al. 2016; Martinez-Aran et al. 2008; Civil Arslan et al 2014; Balanzá-Martínez et al. 2008). Results of this study also confirm cognitive impairments in bipolar patients affecting the speed of motor reactions, which may impair driving performance. Since all patients were on mood-stabilizing medication, most of them on polypharmacy, it is difficult to determine the impact of factors related to the disease and side effects of mood-stabilizing medications on the severity of cognitive deficits. The question relevant for further studies is whether the cognitive dysfunction in BD patients is due to the disease itself or to the side effects of drugs or accumulation of both factors. Cognitive deficits were detected in newly diagnosed patients (Kjærstad et al 2020) as well as in remitted patients with a long history of bipolar disorder. This may indicate that they are a trait feature of the disease. On the other hand, mood stabilizers like lithium are also associated with some cognitive deficits in verbal learning memory and creativity. Moreover, the effect of particular MS drugs on cognitive abilities is likely to be somewhat variable. For example, remitted BP patients taking valproates performed poorer on working memory tasks, but not in other cognitive domains, compared to patients who were on lithium (Muralidharan et al. 2015). For this reason, it is necessary to consider the possibility of cumulative side effects of many drugs used simultaneously as part of polytherapy, which may additionally overlap with the deficits associated with the disease. Although patients taking lithium obtained slightly worse results in some tests, these differences should be interpreted with caution as most of them were also treated with combination of other drugs, which may be a confounding factor. Moreover, the variety of drug combinations made it impossible to compare the results in groups that would be more homogeneous in this respect, but their size would be too small.

Our results do not indicate which level of cognitive decline is critical and may significantly increase the risk of road accidents. This problem has not been sufficiently explained in the results of previous studies and needs further clarification. However, these deficits may vary from patient to patient depending on clinical variables, the number of medications used, and comorbid medical conditions. Therefore, a personalized assessment of driving fitness seems preferable to seeking general recommendations. Standardized driving simulators could be very helpful for such purposes. The other issue is what mood-stabilizing therapeutic strategy for BD patients may be effective to maintain full remission, including enabling safe driving of vehicles. Some authors suggest that that recovery of driving competence should be an integral goal of treatment strategies for psychiatric disorders (Brunnauer et al 2016).

## Limitations of the study

Sixty-five subjects (33 patients and 32 control subjects) participated in the study. The studied group was relatively small, which forces us to formulate cautious conclusions and verify the results after examining a larger group of patients. We applied tests that indirectly indicate the ability to drive motor vehicles (two used in Polish transport psychology and three popular neuropsychological tests). To obtain results closer to the real situations, it would be more appropriate to use driving simulators which would allow for assessing the standard parameters and the quality of reactions while driving a car.

## Conclusions

Patients with BD performed worse in tests dedicated to drivers (COT, ST) and neuropsychological tests in comparison to healthy people. This is especially true of extended response times referred to as second-generation drugs, which may result in an increased risk of road accidents. Therefore, frequent monitoring of cognitive performance in remitted BD patients who drive a car is advisable. Neuropsychological tests, such as the SCWT and TMT may also be useful.

## Data Availability

Scientific data supporting this study are available from Piotr Joachimiak MD Ph.D., Department of Psychiatry, Medical University of Poznań, ul. Szpitalna 27/33, 60-572 Poznań, Poland.

## Abbreviations

BD (BD I or II): Bipolar disorder (bipolar disorder type I or II)
COT: The Cross-over Test; version with free tempo (COT-F) and version with tempo of 50 tasks per minute (COT-50)
DSM-5: Diagnostic and Statistical Manual of Mental Disorders version V
HAM-D: Hamilton Depression Rating Scale
LED: Light-emitting diode
MS (MSD): Mood stabilizer (mood-stabilizing drug)
PJ: Piotr Joachimiak MD PhD
RAVLT: Rey Auditory Verbal Learning Test
SCWT (SCWT-A, SCWT-B): Stroop Color-Word Test (part A, part B)
SD: Standard deviation
SNRI: Serotonin-norepinephrine reuptake inhibitor
SPD: Specialistic Diagnostic Platform
SSRI: Selective serotonin reuptake inhibitor
ST: Signal Test
TMT (TMT-A, TMT-B): Trail Making Test (version A, version B)
YMRS: Young Mania Rating Scale
